# Selecting feeding behavior would enhance feed efficiency in slow-growing free-range broiler

**DOI:** 10.1186/s12711-026-01108-y

**Published:** 2026-11-13

**Authors:** Salomé Chaumont, Sandrine V. Mignon, Elisabeth Le Bihan-Duval, Anne Collin, Frédéric Fagnoul, Elodie Guettier, Julie M. Collet

**Affiliations:** 1 BOA, INRAE, Université de Tours, Nouzilly, 37380 France; 2 Hubbard, Mauguerand, Le Foeil, 22800 France

## Abstract

**Background:**

In the free-range poultry industry, commercial birds have access to outdoor environments where climatic conditions are variable and uncontrolled. However, breeding animals must be selected under confined and controlled conditions for sanitary reasons. These environmental differences can reduce selection efficiency due to genotype-by-environment (G×E) interactions, particularly affecting feed efficiency—a key trait for sustainability that impacts both economic performance and environmental footprint. Therefore, selection based solely on performance in confined conditions may not be optimal for free-range systems. To enhance sustainability in such systems, it is important to identify indoor traits genetically correlated with outdoor feed conversion ratios. This study investigates the potential for integrating feeding behavior traits into selection schemes for slow-growing broiler chickens raised outdoors during summer.

**Results:**

We analyzed various feeding behaviors expressed indoors, including the number of meals, the timing and distribution of intake, and their variability both within and across days, as well as the correlation between these feeding behaviors and temperature. Many traits exhibited moderate to high heritability and were genetically correlated with outdoor feed conversion ratios. However, the correlations between traits were not always favorable: some behavioral traits were positively correlated with feed conversion ratio but they were negatively correlated with each other. To address these interactions, we summarized the **G** matrix of genetic variances and covariances among traits by its first two eigen vectors, g_max_and g_2_, accounting for 74% of the genetic variance in feeding behaviors. The **g**_max_axis included meal frequency, day-to-day variation in early-day intake, and meal number, while **g**_**2**_axis was mainly correlated with traits related to early-day feeding behaviors and their variation. Selection on the gmax axis could enhance feed efficiency and meat quality without compromising carcass yield, unlike selection on g_2_, which would improve feed efficiency at the expense of meat quality and carcass composition.

**Conclusions:**

These findings suggest that incorporating feeding behavior traits, particularly those aligned with gmax, into indoor selection schemes could contribute to enhancing feed efficiency in outdoor environments.

**Supplementary Information:**

The online version contains supplementary material available at https://doi.org/10.1186/s12711-026-01108-y.

## Background

Designed to meet societal expectations for improved animal welfare, free-range systems enable chickens to express natural behaviors and support better overall condition [1]. In France, free-range broilers are mainly produced via the Label Rouge certificate, a seal to guarantee product quality and animal welfare [2, 3]. Label Rouge chickens are approximately twice as expensive to produce as standard broilers (2.5 vs. 1.3 €/kg [2]), largely due to reduced feed efficiency and longer rearing period. Since feed accounts for around 58% of total production costs [2], the feed conversion ratio (FCR, feed intake / weight gain) is a key driver of profitability and sustainability in poultry systems. In Label Rouge production, FCR is substantially higher than in standard production (2.6 vs. 1.7) due to the use of slow-growing genotypes and increased energy expenditure. These factors result in lower daily weight gain (25.8 vs. 54.1 g/day) and higher feed intake over the complete production cycle compared to standard systems (6.9 vs. 3.1 kg [2]). Genetic selection has significantly improved FCR in conventional broilers [4]. However, because most breeding programs are based on raising animals indoors under highly controlled conditions, there are concerns about how well these genetic gains translate to free-range environments, where animals are exposed to more variable climates, diets, and activity levels.

Growth and feed efficiency traits are particularly sensitive to G×E (genotype × environment) effects for at least two reasons [5, 6]. First, heritability estimates of these traits indoors are lower than in free-range conditions (e.g., FCR h² = 0.04 ± 0.03 indoors vs. 0.42 ± 0.09 outdoors [5]). Second, genetic correlations between environmental conditions for residual feed intake, feed intake, and weight gain are significatively lower than 1 [5]. G×E interactions reduce selection accuracy, may reverse animal rankings, and alter genetic correlations with other traits. For example, selection for low FCR improves thigh yield indoors but reduces it outdoors [5], probably as energy needs are 18 to 36% higher when chickens are raised with an outdoor access [7].

To address these challenges, breeding strategies must better account for environmental differences between selection and rearing environments. As breeding schemes include at least two generations of multiplication and two-to-four-line crosses, poultry systems cannot use the commercial animal information to select the nucleus population. Indeed, the information required to inform selection decisions in purebred lines only becomes available with a substantial time lag too late too be used for selecting the corresponding candidates. One option then involves collecting data from “collateral” animals (i.e. related animals such as half- and full-sibs that will not be used in the nucleus population) raised under commercial conditions [8], but this approach is costly, complex, and can reduce selection efficiency up to 30% [9]. Alternatively, selecting animals that are less sensitive to G×E interactions for key production traits may be a more sustainable strategy. Importantly, improving feed efficiency must not be at the expense of final body weight, especially in Label Rouge systems where a specific market weight must be achieved at the regulatory age of 12 weeks. Single‑trait selection focused on feed intake, a trait positively correlated with body weight [10, 11], or selection on body weight itself, may unintentionally lead to deviations of body weight from the targeted standard. Indirect selection of feed efficiency via other traits may help improve performance without significantly altering final body weight.

Feeding behavior has emerged as a promising indirect trait for improving feed efficiency. Chagneau et al. [12] demonstrated in slow-growing caged chickens that providing feed as several meals per day, with short fasting periods in between, reduced FCR by approximately 0.05 compared to continuous access to feed. Hence, intermittent lighting, provoking intermittent meals, reduces FCR [13]. Rapid ingestion of a large quantity of feed could stimulate the upper part of the digestive tract (especially the crop), promoting greater proventricular secretion and gizzard development, which in turn would help improve digestive efficiency [13]. Van der Sluis et al. [14] observed in fast-growing broilers reared in pens that birds with faster early growth performed longer meals, while those with larger body weight gain ate meals of shorter duration. These phenotypic results suggest that meal patterns and frequency could serve as indirect selection criteria for FCR if those traits are heritable.

Feeding behavior is plastic in response to changes in environmental conditions. For instance, reduced feed intake during heat exposure lowers heat production from digestion by 15–40% within 8–24 h [15], thus helping birds to maintain their internal temperature. In practice, farmers often restrict feed access during peak heat. Selecting animals capable of adjusting their feeding behavior under stress may simultaneously improve efficiency and resilience. Howie et al. [16, 17] demonstrated that meal dynamics — including frequency, size, duration, and inter-meal intervals — are biologically conserved across species such as broilers, turkeys, and ducks, and display moderate to high heritability (0.24–0.57). Specific feeding strategies were also genetically associated with favorable FCR values, highlighting their potential across environments. Beyond physiological factors, feeding behavior may also vary with emotional or motivational states. Studies have shown that chronic stress can activate reward pathways, increasing the appeal of feeding [18], while boredom proneness may promote emotional or disorganized eating behavior [19]. These findings suggest that feeding behavior is not solely an efficiency marker but may also serve as an indicator of stress resilience or emotional state, and therefore represents a potential selection criterion in genetic improvement programs.

Our study aimed to assess the potential of incorporating feeding behavior traits into breeding strategies to improve feed efficiency in a slow-growing chicken strain raised under free-range conditions. We focused on a sire-line of a naked-neck breed commonly used in the French Label Rouge sector for its enhanced heat tolerance and reduced performance losses under thermal stress [20, 21]. As in Label Rouge production, the sire line typically contributes 50% or more of the genetic makeup of the final crossbred product, making genetic improvement at the pure-line level directly relevant to commercial performance. We characterized various feeding behavior traits, including meal frequency, meal size, daily intake patterns, behavioral consistency across days, and feeding behavior response to heat. We estimated their heritability and genetic correlations under indoor conditions across two replicate trials of approximately 300 birds. Finally, we identified a profile of feeding behaviors expressed indoors that could be used as indirect selection criteria to improve feed efficiency in free-range systems. This approach also considered potential correlated effects on meat quality and carcass composition, and allowed us to estimate the expected gain in efficiency when selecting on behavioral traits rather than directly on feed efficiency.

## Methods

### Studied population and rearing conditions

This study was conducted using the S77N slow-growing chicken line (Hubbard, France), reared under two housing conditions over two summer periods (2022 and 2023) at the INRAE PEAT experimental unit (doi:https://doi.org/10.15454/1.5572326250887292E12). S77N is a pure line used in Label Rouge production as sire of the commercial product. Detailed descriptions of the experimental setup, including diet composition and environmental conditions, are available in Chaumont et al. [22].

Briefly, 284 in 2022 and 293 in 2023 as-hatched birds were reared entirely indoors until slaughter at 78 days (IN condition). Birds were fed *ad libitum* with a standard starter diet until 27 days, followed by a grower-finisher diet. From day 1 to 56, night-time rest was progressively increased to 8 h, then fixed from day 57 of age (10 p.m. to 6 a.m.). We replicated as much as possible the housing conditions over the two years of experiment (same hatcheries, same dates, etc.), although outside temperatures varied across years. To calculate genetic correlations of traits between rearing conditions, 566 (269 in 2022 and 297 in 2023) collateral birds (half-sibs and full-sibs) were reared in similar conditions (diet, lighting hours, etc.) but had access to a 2800m^2^ outdoor area until slaughter at 82 days (OUT condition), so as to reach similar final body weight in IN and OUT conditions. Pedigrees of all birds were obtained after genotyping the birds using home-made chips.

Despite aiming to maintain constant temperature in IN conditions, technical limits caused variance in indoor temperatures, which in turn may have affected feeding behaviors. Over the period from 40 to 76 days of age, the number of days above 30 °C varied across years (2 in 2022 and 12 in 2023). It is important to note that there are no specific references defining thermal comfort zones for the breed used in our study, but literature indicates that an average temperature around 30 °C negatively affects growth and feed intake in naked neck chickens [21].

### Measurements

#### Growth traits and feed efficiency

Chicks were identified with a wing band at hatch. They were also equipped by a radio frequency identification (RFID) device on the neck at 15 days of age in order to continuously monitor individual body weight and feed consumption thanks to electronic feed stations, as already described [23, 24]. Some birds lost their RFID device and some devices malfunctioned during the rearing period, so that number of birds included in each behavior may vary (Table 1). Using records from the feed stations, we measured each individual’s daily feed intake and daily weight gain to calculate the FCR, defined as the ratio of total feed intake (FI) to total weight gain (WG) over the 40–76 day period.

**Table 1:** Traits measured in the period between 40 and 76 days

Trait	Abbreviation	Description	Measurement Method	N	ATOL
Total feed intake in gram	FI	Total amount of feed consumed in the period	Measured using RFID identification and electronic feeding stations	508	0005507
Total weight gain in gram	WG	Change in body weight over a period of time, divided by the number of days in the period		507	0000988
Feed conversion ratio	FCR	Ratio between the amount of product (milk, meat...) on the amount of feed intake	Calculated as FI/WG	507	0001580
Residual Feed Intake in gram	RFI	Difference between measured feed intake of one animal and predicted feed intake for the group of animals	Difference between feed intake measured by electronic feeding stations and predicted feed intake. Estimated consumption is measured as:where µ is the mean of FI measured, BW^0.75^the metabolic body weight at 76 days of age.	479	0002160
Total number of meals (Log transformed)	MEALNB	Number of distinct sequences of feed intake	Calculated from feeding station visits	508	0000777
Residual Meal Number	RESMEAL	Difference between actual meal number and expected MEALNB	Expected MEALNB was calculated for each bird via a linear regression of MEALNB as a function of age	508	
% of feed intake before 8am	FI8	Proportion of total feed intake before 8am	Based on FI data	508	
% of feed intake after 8pm	FI20	Proportion of total feed intake after 8pm	Based on FI data	508	
% of meals before 8am	MEAL8	Proportion of meals consumed before 8am	Calculated from visit times	507	
% of meals after 8pm	MEAL20	Proportion of meals consumed after 8pm	Calculated from visit times	507	
Log of the variance of residual feed intake	LVRFI	Variability of residual feed intake	Calculated via regression equation	507	
Log of the variance of residual meal number	LVRESMEAL	Variability of residual meals	Calculated by linear regression	507	
Log of the variance of intake before 8am	LVFI8	Variability of early feeding	Logarithm of variance calculation	507	
Log of the variance of intake after 8pm	LVFI20	Variability of late feeding	Logarithm of variance calculation	507	
Log of the variance of intake before 8am	LVMEAL8	Variability of early meal	Logarithm of variance calculation	507	
Log of the variance of intake after 8pm	LVMEAL20	Variability of late meal	Logarithm of variance calculation	507	
Skewness of residual feed intake	SKRFI	Bias toward high or low intake	Skewness on daily data	507	
Skewness of residual meals	SKRESMEAL	Bias towards high or low values of residual meals	Skewness on daily data	507	
Skewness of intake before 8am	SKFI8	Bias toward high or low values of early morning intake	Skewness on daily data	507	
Skewness of intake after 8pm	SKFI20	Bias toward high or low values of late evening intake	Skewness on daily data	507	
Skewness of intake before 8am	SKMEAL8	Bias towards high or low numbers of meals in the early morning	Skewness on daily data	507	
Skewness of intake after 8pm	SKMEAL20	Bias towards high or low numbers of meals in the late evening	Skewness on daily data	507	
Correlation between residual feed intake and temperature	CORRFI	Influence of temperature on feed intake	Pearson correlation with cumulative temperature over 3 days	507	
Correlation between residual meal and temperature	CORRESMEAL	Influence of temperature on residual meal	Pearson correlation with cumulative temperature over 3 days	507	
Correlation between intake before 8am and temperature	CORFI8	Influence of temperature on early intake	Pearson correlation with cumulative temperature over 3 days	507	
Correlation between intake after 8pm and temperature	CORFI20	Influence of temperature on late intake	Pearson correlation with cumulative temperature over 3 days	507	
Correlation between meal before 8am and temperature	CORMEAL8	Influence of temperature on early meal	Pearson correlation with cumulative temperature over 3 days	507	
Correlation between meal after 8pm and temperature	CORMEAL20	Influence of temperature on late meal	Pearson correlation with cumulative temperature over 3 days	507	
pH 15 minutes post mortem	pH15	Meat acidity	Measured on breast with pH meter 15 minutes post-mortem	502	0001684
pH 24 hours post mortem	pHu	Meat acidity	Measured on breast with pH meter 24 hours post-mortem	507	0001684
Breast meat lightness (L*)	L*	Lightness component of meat color measured in the CIE L*a*b* color space	Measured without the skin using a colorimeter	507	0005785
Thigh yield (%)	TY	Weight of the thigh yield normalized by the body weight	Measured as a percentage of live weight at slaughter	507	0001650
Breast yield (%)	BY	Weight of the breast yield normalized by the body weight	Measured as a percentage of live weight at slaughter	504	0001650
Abdominal adipose tissue weight (%)	AFY	Weight of the abdominal adipose tissue normalized by the body weight	Measured as a percentage of live weight at slaughter	502	0001540

N: sample size. ATOL: animal trait ontology for livestock (ATOL) number [53]

Data quality checks were performed on all individual daily feed intake and body weight records prior to analysis. The growth and feed intake curves of each animal were plotted and visually inspected to identify any anomalous trajectories. Raw records flagged as implausible were corrected manually by returning to the raw data or replaced by linear interpolation between the preceding and following valid records. Additionally, a proportion of feed disappearance recorded at the station level could not be attributed to individual animals (close to 5%); this unattributed intake affects the absolute level of FCR estimates but is not expected to introduce systematic bias in genetic analyses, as there is no reason for it to affect certain birds more than others.

#### Feeding behavior

Raw feeder attendance data indicate the start and end times of visits to the feed station. However, a single meal may include multiple visits. To determine the duration of a meal, we used the method described by Howie et al. [25], which enables determining meal duration from the data themselves, so that it evolves with the birds’ age and across systems, feed, etc. Our method to determine meal duration consists of plotting the distribution of the logarithm of the intervals between visits, which takes the form of a mixture of two normal distributions. We took the daily point of intersection of these two distributions and determined that when intervals between two visits were below that threshold, the two visits belonged to the same meal, while high intervals above the threshold were interpreted as different meals. Details on the variation of the limits of one vs. two meals can be found in [Additional file 1, Figure S1]. We recorded feeding behaviors (listed in Table 1) from 40 to 76 days of age. We computed FI and total number of meals (MEALNB) over the same period. These indicators distinguish individuals that consume their feed through fewer meals from those that eat more frequently. To further characterize feeding behaviors, we also examined the distribution of feeding behavior at specific times of the day. We estimated the ability of animals to reduce their ingestion during the hottest hours of the day by calculating the proportion of meals and feed intake occurring during the coldest hours of the day. As digestion produces heat [26], increasing ingestion in coldest hours may increase their temperature control during hot days. Specifically, we computed early morning (meals starting before 8:00am, corresponding to MEAL8 and FI8) and late evening (meals starting after 8:00pm, corresponding to MEAL20 and FI20) behaviors as percentage of meals or feed intake that occurred during these periods over the 40–76 day period. In addition to describing overall feeding behavior, we aimed to evaluate the consistency and the nature of variation in these behaviors over time. To account for the confounding effect of age, as feed intake increases and meal frequency decreases when birds become older [14, 23], we used residual values for variability measures instead of raw daily data for these two parameters. Residual feed intake (RFI_d_) and residual meal number (RESMEAL_d_) were calculated daily as:

1 $$\:{RFI}_{d}={FI}_{d}-\widehat{{FI}_{d}} ,$$

with

$$\:\widehat{{FId}_{d}}=\mu\:+a\times\:{{BW}_{d}}^{0.75}+b\times\:{WG}_{d}$$,

where $\:{FI}_{d}$ is the daily feed intake, $\:\widehat{{FI}_{d}}$ is the estimated feed intake of that day, BW_d_^0.75^ is the metabolic body weight on that day, $\:{WG}_{d}$ is the weight gain from that day compared to the previous day, and µ, * a*, and * b* are the mean and regression coefficients calculated from the overall 40–76 days of age period.

Similarly, daily RESMEAL_d_ followed:

2 $$\:{RESMEAL}_{d}={MEALNB}_{d}-\widehat{{MEALNB}_{d}} ,$$

with $$\:\widehat{{MEALNB}_{d}}=\:\mu\:+\beta\:\:\times\:Age$$,

where $\:\widehat{{MEALNB}_{d}}$ is the estimated number of meals predicted at a particular age, and ​*μ* and $\:\beta\:$are the mean and regression coefficient calculated over the 40–76 days of age period.

Then, we used the daily feeding data (residual number of meals, residual feed intake, proportion of early and late meals, and proportion of early and late consumptions) collected between days 40 and 76 to calculate two types of indicators of individual fluctuations for each behavioral trait:

- Variability, computed as the natural logarithm of the variance of daily individual values (LVRESMEAL, LVRFI, LVMEAL8, LVFI8, LVMEAL20, LVFI20). This quantifies the overall day-to-day fluctuation in behavior, with lower values indicating greater consistency across days.
- Skewness, which provides insight into the direction of individual behavioral fluctuations (SKRESMEAL, SKRFI, SKMEAL8, SKFI8, SKMEAL20, SKFI20). A positive skewness means that the bird exhibited occasional days with particularly high values of the behavior (e.g., feed intake or number of meals). Conversely, a negative skewness indicates occasional days of underperformance of behaviors.

These indicators help identify whether an individual tends to have erratic peaks or persistent patterns in feeding behavior across time, providing a deeper understanding of feeding stability and plasticity, which could indicate resilience and/or robustness [27].

Finally, we investigated whether individual animals adjusted their feeding behavior in response to climatic conditions by calculating, for each bird, the Pearson correlation coefficient between daily behavioral traits and the cumulative maximum temperature over the preceding three days. This three-day window was chosen based on previous studies showing that heat stress affects feed intake and behavior typically beginning after 2 to 3 days of exposure [28, 29]. Specifically, we used the cor function in R version 4.1 [30] (as for all analyses below) to compute these correlations for the traits, assessing how daily behavior covaries with recent heat exposure (CORRESMEAL, CORRFI, CORMEAL8, CORFI8, CORMEAL20, CORFI20).

#### Carcass composition and meat quality

Chicken live weight was measured just before slaughter (at 78 days for the indoor group and at 82 days for the outdoor group), after animals were fasted for 14–17 h. Carcasses were chilled at 4 °C for 24 h and cut to assess carcass composition. Breast meat pH was measured 15 min (pH15) and 24 h (pHu) after slaughter, as specified in Chaumont et al. [22]. The right thigh, right *Pectoralis major*, right *Pectoralis minor*, and abdominal fat were sampled and weighed to calculate breast, thigh, and abdominal fat yields (BY, TY, AFY, respectively) in relation to live body weight, as specified in Chaumont et al. (2025a). Color was measured on the upper ventral side of the *Pectoralis major* by using a spectrocolorimeter (CM 700 D, Konica Minolta Inc, Tokyo, Japan), applying the CIELAB trichromatic system with lightness (L*), redness (a*) and yellowness (b*).

### Statistics analyses

#### Phenotypic analyses

To better understand the influence of sex and year of experiment on feeding behaviors indoors, traits were analyzed using a type III analysis of variance (ANOVA) from the R package car [31] with the following model:

3 $$y_{ijk} = \mu + S_j + Y_k + SY_{jk} + \varepsilon_{ijk},$$

where *y*_*ijk*_ is the trait measured for bird *i*, *µ* is the general mean, *S*_*j*_ is the fixed effect of sex (*j*= male, female), *Y*_*k*_ is the fixed effect of year (k = 2022, 2023), *SY*_*jk*_ is the interaction between sex *j* and year *k*, and *ε*_*ijk*_ is the random error term pertaining to bird *i*. As the assumption of normality of the residuals was not met for MEALNB, the data were log-transformed in order to approach a normal distribution. Multiple comparisons of means were performed by the lsmeans function of the emmeans R package [32], applying the Bonferroni correction method to adjust for multiple testing.

#### Genetics analyses

Genetic parameters were estimated with multivariate models on six standardized (mean = 0, sd = 1) traits according to VCE6.0 [33] recommendations. We chose a set of six traits as a compromise between model convergence (that demands fewer traits) and the search for the best covariance description among traits (that requires more traits). The multivariate animal model was fitted as:

4 $$\:\boldsymbol{y}=\:\mathbf{X}\mathbf{b}+\mathbf{Z}\mathbf{a}+\boldsymbol{\upepsilon\:} ,$$

where $\:\boldsymbol{y}$ is the vector of the six phenotypic observations, $\:\mathbf{b}$ is the vector of fixed effects, $\:\mathbf{a}$ is the vector of additive genetic effects, and $\:\boldsymbol{\upepsilon\:}$ the vector of residual errors. $\:\mathbf{X}$ is the corresponding design matrix for fixed effects and $\:\mathbf{Z}$ the design matrix for random effects. Additive genetic effects were assumed to follow.

**a** ~ *N* (0, **G** ⊗ **A**),

where **A** is the numerator relationship matrix based on a pedigree spanning over 9 generations and 8884 individuals, and **G** is the genetic variance-covariance matrix. Residuals were assumed to follow.

**ε** ~ *N* (0, **R** ⊗ **I**),

where **R** is the 6 × 6 residual variance-covariance matrix between traits and **I** is an identity matrix.

From model (4), we estimated the heritability of each trait in IN condition, as well as the genetic correlations among traits. Because variables appeared in multiple combinations of six, heritability estimates and genetic correlations were obtained more than once. Each heritability and genetic correlation estimates reported here are the mean of the estimates obtained in all models. To calculate these means, we discarded estimates of genetic correlations that showed a standard error above 0.30 to ensure only estimates evaluated in a robust model were included. We further calculated the significance of heritability estimates using a one-tailed Z-test, where the Z-statistic was computed as the ratio of the estimate to its standard error and the corresponding p-value was derived from the standard normal distribution. Significance of genetic correlations were computed using two-tailed Z-tests. Since our objective was to identify feeding behavior traits in IN that could be selected to improve FCR in OUT, feeding behavior traits with a heritability lower than 0.1 in IN were considered not sufficiently heritable for effective selection and were excluded from further analysis. For the traits meeting this heritability threshold, we examined their genetic correlations among themselves and their genetic correlation with FCR in OUT.

Multiple genetic correlations were observed between the studied traits; however, these correlations are often difficult to interpret when analyzed one by one. In order to analyze these multiple traits within a single framework, we considered the genetic variance-covariance matrix, called **G** matrix, which contains the genetic variances of the chosen traits on its diagonal and the genetic covariances among traits off its diagonal [34]. In **G**, we included the genetic variances and covariances from model (4) of the traits with h² > 0.10, since these traits were more likely to show meaningful genetic correlations. These traits were CORRESMEAL, LVFI20, LVFI8, LVRFI, LVMEAL8, LVRESMEAL, FI, MEALNB, FI20, FI8, MEAL20, MEAL8, SKMEAL20 and SKMEAL8. The eigenvalues and eigenvectors of the **G** matrix were calculated using the eigen() function [35] in R, to estimate the number of axes and dimensions that actually vary in the genetic space of the studied traits. We kept the first two eigenvectors (**g**_**max**_ and **g**_**2**_) for further analyses.

To estimate the heritability of **g**_**max**_ and **g**_**2**_, and their genetic correlations with outdoor performance traits, we computed individual-level scores for each animal by projecting their phenotypic values onto each eigenvector — that is, by calculating the weighted sum of each animal’s phenotypic values using the eigenvector coefficients as weights. These scores represent each animal’s position along each genetic axis and can be treated as quantitative phenotypes. They were then included in the model (4) alongside outdoor performance traits to estimate variance components and genetic correlations.

Regarding the possible strategies to improve FCR in production conditions (i.e. FCR OUT), we compared three scenarios: (i) the currently used selection method, where selection criterion is FCR IN, (ii) a direct selection method, where FCR OUT would be selected in a free-range environment, (iii) an indirect selection on behaviors in IN (scenario A). Relative efficiencies of the selection method we propose (A) was compared to either methods (i) or (ii) (scenario B) as the ratio of expected gains to selection in both methods according to the Eq. (5) adapted from the calculation of the response to indirect selection according to:

5 $$\:\frac{\sqrt{{h}_{{SC}_{A}}^{2}}\times\:{r}_{g}({SC}_{A},\:FCR\:OUT)}{\sqrt{{h}_{{SC}_{B}}^{2}}\times\:{r}_{g}({SC}_{B},\:FCR\:OUT)} ,$$

where $\:{SC}_{A}$ and $\:{SC}_{B}$ are the selection criteria of scenarios A and B, with method A corresponding to indirect selection on behaviors in IN, $\:{h}_{{SC}_{A}}^{2}$and $\:{h}_{{SC}_{B}}^{2}$, the heritability of the selection criteria of scenario A and B, respectively, and $\:{r}_{g}({SC}_{A},\:FCR\:OUT)$ and $\:{r}_{g}\left({SC}_{B},\:FCR\:OUT\right)\:$the genetic correlations between FCR OUT and the traits selected in scenario A and B, respectively.

## Results

### Phenotypic analyses

Tables 2 , 3, and 4 compare the expression of feeding behaviors according to sexes, year, and the interaction effect of sex and year. Birds showed similar FCR (2.5 vs. 2.6) and feed intake (95 g/day) both years, but they ingested more meals in 2022 than in 2023 (626 vs. 585 respectively, Table 2). This suggests environmental differences across years affected feeding behavior without affecting efficiency. A significant year effect was observed for all variables related to feeding behavior distribution. Birds in 2022 consumed a greater proportion of their feed in the early morning than birds in 2023, and a lower proportion in the late evening than birds in 2023 (Table 2). Males and females differed in all daily traits; females consumed more meals (636 vs. 575), had a lower FI (87 vs. 104 g/day) and a higher FCR than males (2.76 vs. 2.32). Early morning and late evening consumption did not significantly vary between sexes, although there was a small interaction between sex and year for MEAL20 (Table 2).

**Table 2:** Least-square means of feeding behaviors. according to sex (F and M), year (2022-2023), and their interactions

		FCR	FI	MEALNB	FI8	FI20	MEAL8	MEAL20
	N	497	497	508	508	508	508	508
Sex	F	2.76	87	636	0.216	0.118	0.202	0.122
	M	2.32	104	575	0.218	0.120	0.208	0.125
	SE	0.01	1	8.84	0.002	0.002	0.001	0.001
	P-value	< 0.001	< 0.001	0.001	0.557	0.610	0.080	0.683
Year	2022	2.52	95	626	0.239	0.088	0.228	0.096
	2023	2.56	95	585	0.195	0.150	0.183	0.152
	SE	0.01	1	9	0.002	0.002	0.001	0.001
	P-value	0.083	0.342	0.021	< 0.001	< 0.001	< 0.001	< 0.001
Sex × Year^1^	F - 2022	2.69	85	657	0.238	0.087	0.225	0.097
	M - 2022	2.35	106	595	0.240	0.089	0.230	0.095
	F - 2023	2.83	88	615	0.194	0.148	0.179	0.148
	M - 2023	2.28	101	554	0.196	0.151	0.187	0.155
	SE	0.02	1	13	0.002	0.002	0.002	0.002
	P-value	< 0.001	0.010	0.997	0.935	0.840	0.519	0.045

FCR: feed conversion ratio (feed intake/weight gain, 40–76 days); FI: average daily feed intake (g/day, 40–76 days);MEALNB: total number of meals; FI8 and FI20: percentage of feed intake before 8am and after 8pm; MEAL8 andMEAL20: percentage of meals before 8am and after 8pm. N: number of individuals with records for each trait. P-value: Significance of the sex/ year/ interaction effects. SE: standard error of the least-square means

**Table 3:** Least-square means of within-individual variability and symmetry of feeding behavior according to sex (F and M), year (2022–2023), and their interactions

* N*		LV RFI	LVRES MEAL	LV FI8	LV FI20	LV MEAL8	LV MEAL20	SK RFI	SKMEAL RES	SKFI8	SKFI20	SK MEAL8	SK MEAL20
		507	507	507	507	507	507	507	507	507	507	496	507
Sex	F	18.98	2.20	-5.55	-5.77	-5.74	-6.09	-0.11	0.16	0.32	0.44	0.41	0.47
	M	19.51	2.29	-5.28	-5.61	-5.38	-5.91	-0.51	0.22	0.55	0.44	0.69	0.68
	SE	0.03	0.03	0.04	0.03	0.03	0.02	0.04	0.03	0.05	0.04	0.04	0.03
	*P-value*	*< 0.001*	*0.014*	*< 0.001*	*< 0.001*	*< 0.001*	*0.003*	*< 0.001*	*0.296*	*0.033*	*0.189*	*0.001*	*0.001*
Year	2022	19.10	2.31	-5.48	-5.95	-5.52	-6.26	-0.32	0.23	0.41	0.62	0.56	0.78
	2023	19.39	2.18	-5.34	-5.44	-5.59	-5.74	-0.30	0.16	0.47	0.26	0.53	0.37
	SE	0.03	0.03	0.04	0.03	0.03	0.02	0.04	0.03	0.05	0.04	0.04	0.03
	*P-value*	*< 0.001*	*0.121*	*0.008*	*< 0.001*	*0.293*	*< 0.001*	*0.515*	*0.223*	*0.617*	*0.001*	*0.391*	*< 0.001*
Sex × Year^1^	F − 2022	18.78	2.25	-5.64	-6.06	-5.71	-6.34	-0.14	0.20	0.29	0.57	0.44	0.67
	M − 2022	19.42	2.38	-5.33	-5.84	-5.34	-6.19	-0.50	0.26	0.52	0.67	0.69	0.89
	F − 2023	19.18	2.16	-5.45	-5.48	-5.78	-5.84	-0.08	0.13	0.35	0.31	0.38	0.27
	M − 2023	19.60	2.20	-5.23	-5.39	-5.41	-5.63	-0.52	0.19	0.59	0.22	0.69	0.46
	SE	0.04	0.04	0.05	0.04	0.04	0.03	0.06	0.04	0.08	0.06	0.05	0.05
	*P-value*	*0.005*	*0.249*	*0.353*	*0.094*	*0.967*	*0.362*	*0.519*	*0.989*	*0.928*	*0.082*	*0.522*	*0.782*

LVRFI: log variance of residual feed intake; LVRESMEAL: log variance of residual meals; LVFI8 and LVFI20: log variance of feed intake before 8am and after 8pm; LVMEAL8 and LVMEAL20: log variance of meal frequency before 8am and after 8pm; SKRFI: skewness of residual feed intake; SKRESMEAL: skewness of residual meals; SKFI8 and SKFI20: skewness of feed intake before 8am and after 8pm; SKMEAL8 and SKMEAL20: skewness of meal frequency before 8am and after 8pm. N: number of individuals with records for each trait. P-value: Significance of the sex/ year/ interaction effects. SE: standard error of the least-square means

**Table 4:** Mean correlations between temperature and feeding behaviors according to sex (F and M), year (2022–2023), and their interactions

N		CORRFI	CORRESMEAL	CORFI8	CORFI20	CORMEAL8	CORMEAL20
		507	507	507	507	507	507
Sex	F	0.03	-0.15	-0.05	0.05	-0.05	0.03
	M	0.10	-0.18	-0.03	0.05	-0.05	0.00
	SE	0.01	0.02	0.01	0.01	0.01	0.01
	*P-value*	0.090	0.845	0.447	0.147	0.101	0.002
Year	2022	0.20	-0.29	-0.13	-0.04	-0.18	-0.09
	2023	-0.07	-0.03	0.05	0.14	0.09	0.12
	SE	0.01	0.02	0.01	0.01	0.01	0.01
	*P-value*	< 0.001	< 0.001	< 0.001	< 0.001	< 0.001	< 0.001
Sex × Year^1^	F − 2022	0.18	-0.29	-0.14	-0.02	-0.16	-0.05
	M − 2022	0.22	-0.30	-0.12	-0.06	-0.20	-0.13
	F − 2023	-0.11	0.00	0.03	0.12	0.06	0.10
	M − 2023	-0.02	-0.05	0.06	0.15	0.11	0.13
	SE	0.02	0.02	0.02	0.02	0.02	0.02
	*P-value*	0.194	0.295	0.714	0.071	0.010	0.002

CORRFI: correlation between residual feed intake and temperature; *CORRESMEAL*: correlation between residual meal number and temperature; CORFI8 and CORFI20: correlations between feed intake and temperature before 8am and after 8pm; CORMEAL8 and CORMEAL20: correlations between meal number and temperature before 8am and after 8pm. N: number of individuals with records for each trait. P-value: Significance of the sex/ year/ interaction effects. SE: standard error of the least-square means

Males showed significantly greater variation than females in all measured behavioral traits (Table 3). This additional variance in males resulted in daily values of behavioral traits that were more skewed towards low values (SKRFI), or high values (SKFI8, SKMEAL8, SKMEAL20), whilst skewness towards high or low values were comparable between sexes for SKMEALRES and SKFI20 (Table 3). There was more variation across days in most individual feeding behaviors in the second year of experiment than in the first (Table 3). Nevertheless, it was in 2022 that birds showed the strongest tendency to exhibit higher positive skewness in their late feeding behaviors (SKFI20 and SKMEAL20). The Sex × Year interaction was only significant for LVRFI, indicating that females in 2022 exhibited a lower variability in feed intake compared to other groups (18.8 for females in 2022 vs. 19.20 and above for other groups).

Most phenotypic correlations between feeding behaviors and ambient temperature were low (i.e., < 0.10, Table 4), indicating a limited direct influence of temperature on feeding patterns. Nevertheless, birds showed a decrease in residual meal number with increasing temperature, as indicated by their negative CORRESMEAL (Table 4). The relationship between feeding behavior and temperature changed across years. In 2022, several traits showed moderate correlations with temperature, including CORRFI (0.20), CORFI8 (–0.13), CORRESMEAL (–0.29), and CORMEAL8 (–0.18), indicating a tendency to reduce morning consumption and the number of meals, while increasing overall feed intake during hot conditions. By contrast, in 2023, correlations between temperature and early morning and late evening feeding behaviors became positive (CORFI8: 0.05, CORFI20: 0.14, CORMEAL8: 0.09, CORMEAL20:0.12), suggesting a behavioral adjustment toward feeding during cooler hours of the day on hot days. The shift in a higher proportion of meals consumed in cooler hours was even stronger in males, as shown by a significant sex × year effect of CORMEAL8 and CORMEAL20.

### Heritability of feeding behavior traits

Table 5 shows phenotypic variances and heritability estimates of feeding behavior traits that could be used as selection criteria. Traits related to meal, and their variability, tended to show higher heritability than those linked to feed intake and how it varied (Table 4). MEALNB exhibited the highest heritability (h² = 0.55 ± 0.08), followed by MEAL8 and MEAL20 (Table 5). Feed intake traits still all displayed significant heritability estimates (Table 5​).

**Table 5:** Phenotypic and genetic parameters of feeding behaviors

Traits^1^	Vp	h²	*P*-value (h^2^)	*r*_g_ FCR	*P*-value (*r*_g_)
CORFI8	0.21	0.09 ± 0.05	0.036	-0.12 ± 0.30	0.689
CORFI20	0.23	0.06 ± 0.04	0.067	NA^2^	NA^2^
CORMEAL8	0.21	0.08 ± 0.05	0.055	-0.55 ± 0.23	0.017
CORMEAL20	0.20	0.08 ± 0.05	0.055	-0.24 ± 0.30	0.424
CORRESMEAL	0.39	0.18 ± 0.07	0.005	0.93 ± 0.06	< 0.001
CORRFI	0.24	0.11 ± 0.06	0.033	-0.14 ± 0.06	0.020
FCR	253,493	0.07 ± 0.04	0.040	0.77 ± 0.15	< 0.001
FI	8,640,873	0.12 ± 0.05	0.008	0.56 ± 0.26	0.031
FI8	0.24	0.28 ± 0.08	< 0.001	-0.32 ± 0.20	0.110
FI20	0.14	0.22 ± 0.08	0.003	0.36 ± 0.12	0.003
LVFI8	541,135	0.21 ± 0.07	0.001	-0.35 ± 0.29	0.227
LVFI20	569,091	0.15 ± 0.05	0.001	-0.59 ± 0.10	< 0.001
LVMEAL8	55,565	0.21 ± 0.06	< 0.001	-0.51 ± 0.18	0.005
LVMEAL20	599,965	0.11 ± 0.05	0.014	-0.31 ± 0.26	0.233
LVRESMEAL	2,249,207	0.31 ± 0.06	< 0.001	0.46 ± 0.15	0.002
LVRFI	1,925,171	0.16 ± 0.06	0.004	0.28 ± 0.16	0.080
MEAL8	0.22	0.39 ± 0.08	< 0.001	-0.38 ± 0.17	0.025
MEAL20	0.14	0.39 ± 0.10	< 0.001	0.24 ± 0.18	0.182
MEALNB	67,660,451	0.55 ± 0.08	< 0.001	0.49 ± 0.13	< 0.001
SKFI8	1.28	0.06 ± 0.03	0.023	NA^2^	NA^2^
SKFI20	1.05	0.02 ± 0.02	0.159	NA^2^	NA^2^
SKMEAL8	0.61	0.16 ± 0.06	0.004	0.15 ± 0.11	0.173
SKMEAL20	1.05	0.22 ± 0.07	< 0.001	-0.25 ± 0.22	0.256
SKRESMEAL	0.65	0.05 ± 0.04	0.106	NA^2^	NA^2^
SKRFI	0.94	0.03 ± 0.03	0.159	-0.57 ± 0.26	0.028
**g**_**max**_	2.94	0.51 ± 0.06	< 0.001	0.43 ± 0.18	0.017
**g**_**2**_	0.05	0.55 ± 0.08	< 0.001	-0.49 ± 0.15	0.001

Vp: Phenotypic variance. h²: Heritability estimates. rg FCR: genetic correlations between feeding behavior traits and FCR OUT. Heritabilities and rg are expressed ± standard error and P-values are calculated from Z-test

CORFI8 and CORFI20: correlations between feed intake before 8am and after 8pm and temperature; CORMEAL8 and CORMEAL20: correlations between meal number before 8am and after 8pm and temperature; CORRESMEAL: correlation between residual meal number and temperature; CORRFI: correlation between residual feed intake and temperature; FCR: feed conversion ratio (feed intake/weight gain, 40–76 days); FI: average daily feed intake (g/day, 40–76 days); FI8 and FI20: percentage of feed intake before 8am and after 8pm; LVFI8 and LVFI20: log variance of feed intake before 8am and after 8pm; LVMEAL8 and LVMEAL20: log variance of meal frequency before 8am and after 8pm; LVRESMEAL: log variance of residual meals; LVRFI: log variance of residual feed intake; MEAL8 and MEAL20: percentage of meals before 8am and after 8pm; MEALNB: total number of meals (log scale);; SKFI8 and SKFI20: skewness of feed intake before 8am and after 8pm; SKMEAL8 and SKMEAL20: skewness of meal frequency before 8am and after 8pm; SKRESMEAL: skewness of residual meals; SKRFI: skewness of residual feed intake, **g**_**max**_ and **g**_**2**_ the first two eigenvectors of the **G** matrix

NA indicates cases where the estimation of genetic parameters did not converge

The variance of daily meal and intake patterns, expressed as log-transformed variances, showed moderate but significant heritability, ranging from 0.11 to 0.31 (Table 5). Skewness traits generally exhibited low and unsignificant heritability, indicating limited genetic control over asymmetry in feeding behavior, with the exception of SKMEAL20, SKMEAL8, and SKFI8 (Table 5).

The heritability of traits relating to the correlation between feeding behavior and temperature was generally low (ranging from 0.06 to 0.18, Table 5​). The correlation between residual meal number and temperature showed the highest heritability (0.18 ± 0.07). This suggests a limited genetic influence on temperature-related behavioral adjustments under indoor rearing conditions.

### Genetic correlations between feeding behaviors in IN and FCR in OUT

We examined the genetic correlations between feeding behavior traits in IN and FCR in OUT to assess whether selecting for feeding behavior traits in IN could improve (i.e., decrease) FCR in OUT conditions. We observed significant positive correlations between FCR OUT and CORRESMEAL, FI, MEALNB, LVRESMEAL, and FI20 (Table 5). Conversely, the genetic correlation between feeding behavior traits in IN and FCR in OUT was significantly negative for LVFI20, SKRFI, CORMEAL8, LVMEAL8, MEAL8, and CORRFI (Table 5). Nevertheless, feeding behaviors also vary between each other and those correlations need be taken into account to fully grasp the effect of feeding behavior selection on feed efficiency.

### Genetic correlation among feeding behavior traits to better understand the effects of multi-trait selection on FCR

Table 6  shows the complex picture of the genetic correlations among feeding behavior traits, and whether these correlations would result in joint or antagonist effect on FCR OUT. Several behaviors (FI8, MEAL8, LVFI20, LVFI8, LVMEAL20, LVMEAL8, SKMEAL20, CORRFI) showed genetic correlations suggesting that selecting for higher values of these traits could improve feed efficiency. Other behaviors (LVRFI, CORRESMEAL, SKMEAL8) showed more complex relationships: although positively correlated with FCR, these traits were indirectly associated with lower FCR via their genetic correlations with traits favorably correlated to FCR. It is therefore essential to implement a multi-trait selection strategy that takes these correlations into account​.

**Table 6:** Genetic correlations among indoor feeding behaviors

	LV RFI	CORRES MEAL	SK MEAL8	FI	FI20	MEAL 20	LVRES MEAL	MEAL NB	FI8	MEAL8	SK MEAL20	LV FI20	LV MEAL20	LV MEAL8	LV FI8	COR RFI
LVRFI		0.72		0.46								-0.08				
CORRESMEAL			0.28	0.29					-0.26	-0.06		NA				NA
SKMEAL8	-0.16				0.02				-0.68	-0.56			NA			
FI			-0.28		0.44	0.35	0.1	0.43		-0.01	-0.14	-0.19	-0.92	-0.27	-0.36	-0.51
FI20	-0.37	-0.12				0.73	0.11	0.37	0.07	0.06	-0.8	0.13	NA	-0.67	-0.39	
MEAL20	-0.25	-0.06	-0.52				0.11	0.26	-0.46	-0.33	-0.78	-0.01		-0.48	-0.55	
LVRESMEAL	-0.12	-0.03	-0.15					0.93	-0.63	-0.81	-0.45	-0.63	-0.99	-0.84	-0.7	-0.55
MEALNB	-0.3	-0.33	-0.21						-0.66	-0.88	-0.63	-0.86	-0.88	-0.89	-0.77	-0.46
FI8	0.58			0.27						0.75	0.23	0.01	0.36	0.43	0.38	NA
MEAL8	0.23										0.45	0.07	0.63	0.61	0.28	0.15
SKMEAL20	0.59	0.28	0.79									0.3	0.73	0.82	0.67	0.9
LVFI20		NA	0.54										0.8	0.68	0.75	0.73
LVMEAL20	0.71	0.95	NA			0.44								0.8	0.74	0.87
LVMEAL8	0.25	0.57	0.51												0.86	0.61
LVFI8	0.48	0.34	0.68													0.65
CORRFI	0.5	NA	0.74		0.19	0.12										

Favorable correlations (above the diagonal) occur when the genetic correlation between two traits aligns with their relationship to FCR: both traits have the same sign correlation with FCR and are positively correlated with each other or they have opposite signs with FCR and are negatively correlated with each other. Unfavorable correlations (below the diagonal) occur when this pattern is reversed—traits with the same sign correlation to FCR but negatively correlated, or with opposite signs but positively correlated—potentially complicating selection for feed efficiency

LVRFI: log variance of residual feed intake; CORRESMEAL: correlation between residual meal number and temperature; SKMEAL8: skewness of meal frequency before 8am; FI: total feed intake; FI20: percentage of feed intake after 8pm; MEAL20: percentage of meals after 8pm; LVRESMEAL: log variance of residual meals; MEALNB: total number of meals (log scale); FI8: percentage of feed intake before 8am; MEAL8: percentage of meals before 8am; SKMEAL20: skewness of meal frequency after 8pm; LVFI20: log variance of feed intake after 8pm; LVMEAL20 and LVMEAL8: log variance of meal frequency after 8pm and before 8am; LVFI8: log variance of feed intake before 8am; CORRFI: correlation between residual feed intake and temperature

NA indicates cases where the estimation of genetic parameters did not converge

**Fig. 1:**
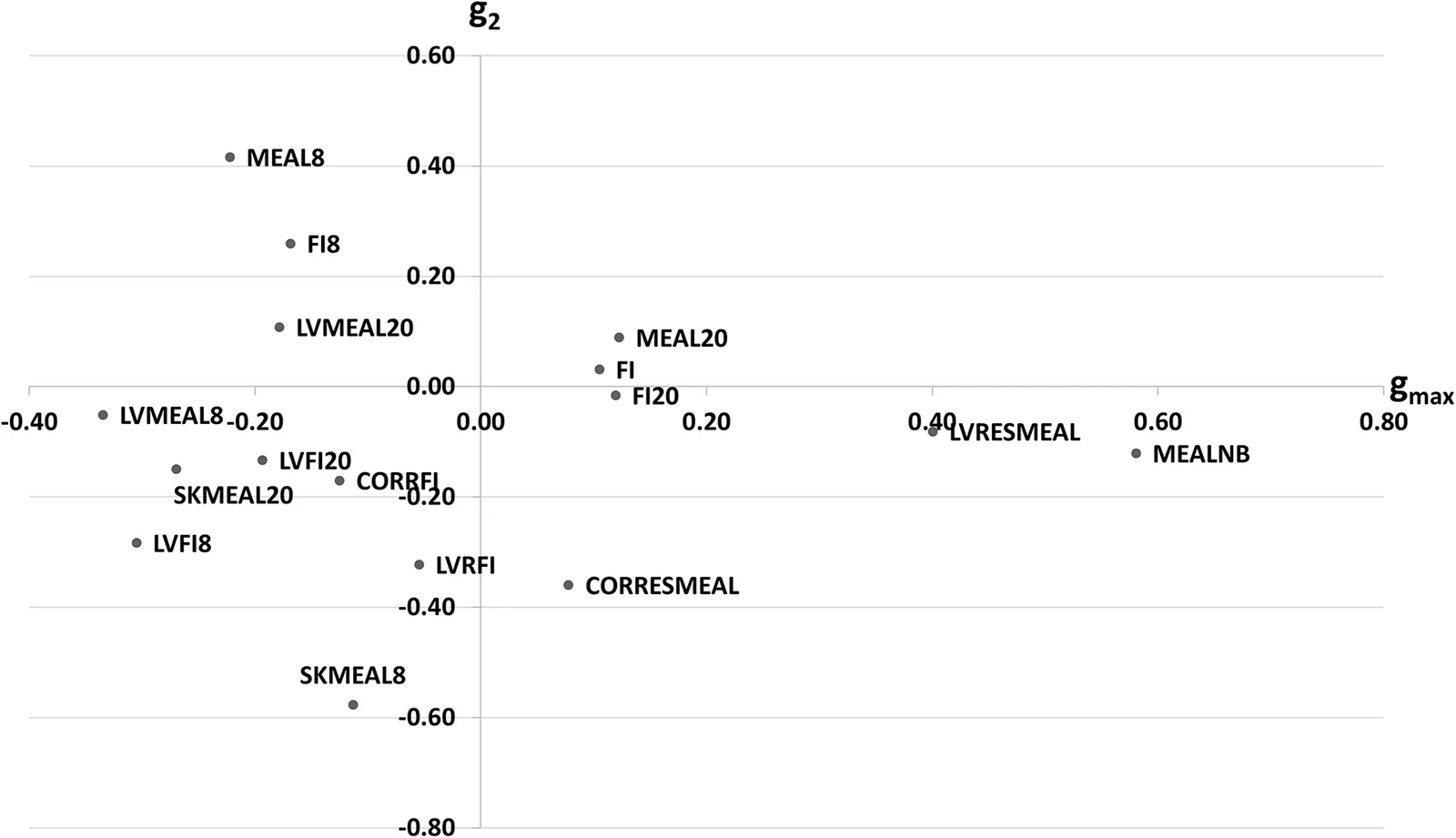
Feeding behavior traits contributions to the first two G matrix axes. CORRESMEAL: correlation between residual meal number and temperature; CORRFI: correlation between residual feed intake and temperature; FCR: feed conversion ratio between 40 and 76 d of age; FI: average daily feed intake (g/d, 40–76 d); FI8 and FI20: percentage of feed intake before 8am and after 8pm; LVFI8 and LVFI20: log variance of feed intake before 8am and after 8pm; LVMEAL8 and LVMEAL20: log variance of meal frequency before 8am and after 8pm; LVRESMEAL: log variance of residual meals; LVRFI: log variance of residual feed intake; MEAL8 and MEAL20: percentage of meals before 8am and after 8pm; MEALNB: total number of meals (log scale); SKMEAL8 and SKMEAL20: skewness of meal frequency before 8am and after 8pm

Using the **G** matrix framework summarizes this complexity. The first two axes of the **G** matrix explained 74% of the genetic variance in our data (56% for the first axis **g**_**max**_ and 18% for the second axis **g**_**2**_). The highest contributions to **g**_**max**_ were from MEALNB, LVRESMEAL, LVMEAL8, LVFI8, SKMEAL20, and MEAL8, while **g**_**2**_ was correlated with SKMEAL8, MEAL8, CORRESMEAL, LVRFI, LVFI8, and FI8 (Fig. 1). Running animal models on **g**_**max**_ and **g**_**2**_ axes projection in the population, we saw that both traits were heritable (Table 5). Moreover, **g**_**max**_ was genetically positively correlated with FCR measured outdoors, while **g**_**2**_ was negatively correlated with FCR OUT (Table 5).

### Genetic gain on FCR in OUT depending on the traits selected and the selection method

Heritability of FCR was estimated at 0.42 ± 0.09 outdoors, but only 0.07 ± 0.03 indoors. The genetic correlation of FCR IN and FCR OUT was estimated at 0.77 ± 0.15. Taking into account these genetic parameters, direct selection of FCR in OUT is still the most efficient method. The expected genetic gain on FCR in OUT when selecting on indoor **g**_**max**_ or **g**_**2**_ would represent, respectively, 47% and 56% of the gain achieved through direct selection on FCR OUT. These results highlight the potential of selecting on these proxy traits in contexts where direct phenotyping outdoor FCR is not feasible. However, the expected genetic gain on FCR in OUT would be 1.99 and 3.36 times higher, respectively, when selecting on indoor **g**_**max**_ or **g**_**2**_ compared to selecting on indoor FCR, as it is currently practiced. This highlights the stronger predictive power of feeding behavior for outdoor performance and its potential relevance for improving feed efficiency in extensive conditions.

### Potential trade-offs of selecting for feeding behavior traits

**Fig. 2:**
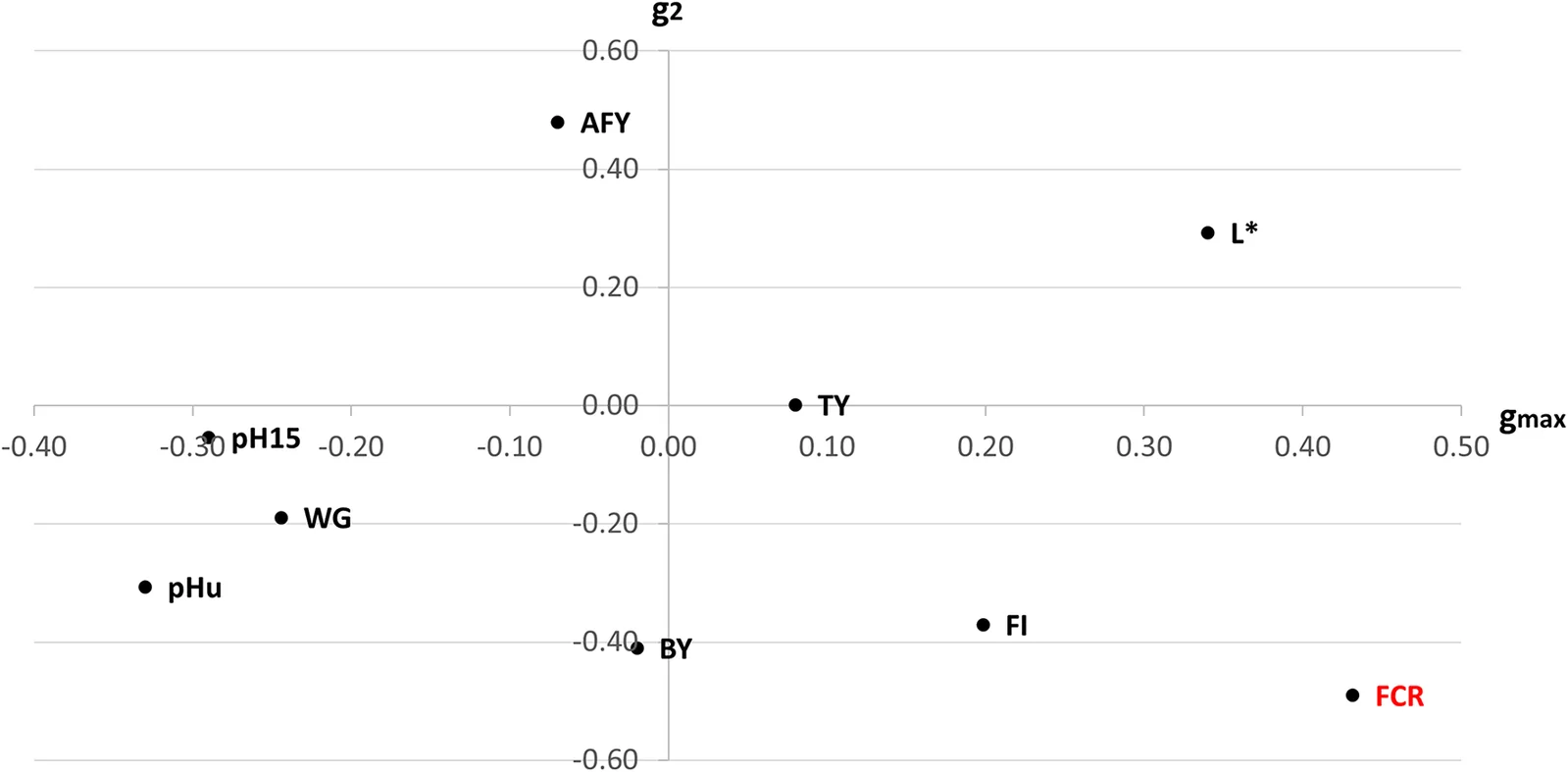
Genetics correlations of growth, feed intake, feed conversion ratio, meat quality and carcass composition traits to the first two G matrix axes. AFY: abdominal fat yield, expressed as a percentage of live weight at slaughter; BY: breast yield, expressed as a percentage of live weight at slaughter; FCR: feed conversion ratio between 40 and 76 days of age, calculated as total feed intake divided by total weight gain over the period; FI: average daily feed intake (g/d, 40–76 d); L*: breast meat lightness (L* value in CIE Lab color space), measured without skin using a colorimeter; pH15: breast meat pH measured 15 min post mortem; pHu: breast meat ultimate pH measured 24 h post mortem; TY: thigh yield, expressed as a percentage of live weight at slaughter; WG: average daily weight gain between 40 and 76 days of age (g/d)

Changes in weight gain and feed intake under selection for **g**_**max**_ are expected to be modest and difficult to predict, given the uncertainty surrounding the genetic correlations between **g**_**max**_ and weight gain (r_g_ = -0.24 ± 0.28), and between **g**_**max**_ and feed intake (r_g_ = 0.20 ± 0.17). The genetic correlations between **g**_**2**_ and feed intake (r_g_ = -0.37 ± 0.18) and **g**_**2**_ and weight gain (r_g_ = -0.19 ± 0.26) suggest that selecting for high **g**_**2**_ would improve FCR by decreasing FI, with unclear effects on WG (Fig. 2).

Selecting for a lower **g**_**max**_ would not change carcass composition (BY: r_g_ =-0.02 ± 0.06; TY: r_g_ = 0.08 ± 0.05; AFY: r_g_ = -0.07 ± 0.11) but would increase pH15 (r_g_ = -0.29 ± 0.06) and pHu (r_g_ = -0.33 ± 0.10) in OUT, which could favorably affect meat quality in this genotype in which meat pH is typically low. However, selecting for a higher **g**_**2**_ in IN would have an impact on carcass composition by decreasing BY (r_g_ = -0.45 ± 0.10) and increasing AFY (r_g_ = 0.42 ± 0.11). Selection for a higher **g**_**2**_ would also decrease meat quality by increasing L* (r_g_ = 0.34 ± 0.13) and decreasing pHu (r_g_ = -0.27 ± 0.15). These results suggest that while **g**_**max**_ selection could simultaneously enhance feed efficiency and meat quality, **g**_**2**_ selection would decrease meat quality and yields.

## Discussion

The objective of this study was to evaluate the potential for integrating feeding behavior traits into genetic selection programs to indirectly select for FCR outdoors while selection is done indoors. Feeding behavior traits expressed indoors displayed sexual dimorphism and interannual variation, highlighting their plastic expression. Most of the studied feeding behaviors showed moderate to high heritability, alongside substantial genetic correlations with FCR. Importantly, we demonstrated that selecting for a combination of feeding behavior traits indoors would yield a higher genetic gain in FCR outdoors compared to the current practice of selecting for FCR indoors. Moreover, this approach would not only improve feed efficiency but would also enhance important production traits such as breast yield and meat quality. Our results suggest that incorporating feeding behavior into genetic selection programs offers a promising strategy to optimize FCR and increase overall production efficiency in poultry systems.

### Phenotypic analyses

This study highlights the influence of sex and environmental conditions on the feeding behavior of chickens raised indoors. Males are known to be on average more feed-efficient than females [11, 36], and our study confirmed this. We also showed that males had more erratic feeding behaviors during the rearing period than females. This consistency in behavioral expression in females suggests that they are more robust and predictable throughout the rearing cycle, which is also an important advantage from a farmer’s perspective. However, both sexes showed limited behavioral adjustment to temperature, reducing only their number of meals in warmer conditions.

The interannual variations observed in this study may highlight behavioral plasticity in response to thermal conditions. In 2023, a warmer year than in 2022, birds ate less often, but proportionally more in the evening and less in the morning than in 2022. Interestingly, birds in 2023 shifted their feeding towards cooler hours of the day in warmer days, suggesting an adjustment of feeding patterns to cope with elevated temperatures. Such behavioral adjustments, aimed at minimizing the thermogenic effect of digestion during heat stress, are well-documented in poultry [15, 28]. By contrast, birds responded in an opposite manner to temperature in 2022: they increased their feed intake, and took a lower proportion of their meals in cooler hours when the ambient temperature was higher. We hypothesize that thermal stress levels may have been insufficient in 2022, a year with fewer and milder heatwaves (see details of climatic conditions here [22]), to elicit pronounced behavioral adaptations. Importantly, high temperatures did not occur at the same stage of growth in both years: around day 50 in 2022 and after day 60 in 2023 (see details in [22]). The stronger correlation between temperature and feed intake observed in 2022 may therefore reflect not only the effect of temperature itself, but also the age at which birds were exposed to it. Disentangling these two factors would require more controlled experimental conditions [22]. Moreover, the “naked neck” genotype used in this study is known for its superior heat tolerance [20, 37, 38], as a 20–40% reduction in feather coverage enables more effective heat dissipation. This genetic factor may have mitigated the need for intense behavioral adaptations by reducing the thermal load experienced. Nevertheless, the thermal comfort zones for this genotype remain poorly defined. Their optimal temperature is likely higher than the 18–21 °C generally recommended for fully feathered birds [39, 40]. Establishing such thresholds is essential for interpreting observed responses and adapting husbandry practices in the face of ongoing climate change. Altogether, these results reveal complex behavioral plasticity to thermal constraints and metabolic demands, confirming that feeding behavior is a relevant lever for understanding, and potentially improving, feed efficiency in poultry production systems.

### Heritable indoor feeding behavior traits

Relatively few estimates of genetic parameters for feeding behavior are available in the literature. The heritability of the number of meals, estimated at 0.55 under indoor conditions in our study, is similar to those reported by Howie et al. [16] in broiler chickens and by Li et al. [41] and Chapuis et al. [42] in Pekin ducks. To our knowledge, there are no other published estimates in poultry of the heritability of the other feeding behaviors explored in this study. Other feeding behavioral traits mentioned in the earlier-mentioned existing studies also show moderate heritability, such as meal duration (h² = 0.30 to 0.65) or feeding rate (h² = 0.38 to 0.59 [25, 41, 42]).

The temporal distribution of meals and intake throughout the day (early or late) and its stability in time emerges as a particularly relevant trait. This distribution of meals during the day captures individual differences in feeding patterns related to the timing of intake, which could be linked to metabolic rhythms or adaptation to environmental conditions. For instance, in our study, MEAL8 and MEAL20 showed higher heritability than the corresponding feed intake quantities consumed during these periods (FI8 and FI20). This suggests that the genetic control of when animals choose to eat during the day might be stronger than that of how much they eat at those times. Such patterns of meal timing could therefore represent meaningful behavioral traits for selection, potentially influencing feed efficiency by optimizing digestion and metabolism according to circadian rhythms.

Variability and asymmetry in all types of behaviors have attracted increasing interest in recent years, particularly for their potential as indicators of robustness. They may reflect an animal’s ability to maintain consistent patterns or to adapt to changes, hence its robustness and/ or resilience. Bédère et al. [27] similarly used distributional properties of longitudinal production records, including the variance, autocorrelation, and asymmetry of weekly deviations in egg production, as indicators of resilience in laying hens. Despite generally low heritability estimates for these indicators, the variance of deviations showed favorable genetic correlations with total egg production, suggesting that resilience could be improved without compromising productivity. In our study, we observed similar trends, with significant correlations between the variation of some feeding behavior traits and FCR OUT (notably LVFI20, LVMEAL8, LVRESMEAL, SKRFI). Nevertheless, we showed relatively higher heritability estimates of our variance-based traits (0.11 to 0.31) compared to previous studies (0.02 to 0.12), indicating promising potential for selection aimed at improving feed efficiency. Consistent with Bédère et al. [27], heritability of asymmetry-related traits was generally low (< 0.10). The exceptions, SKMEAL8 and SKMEAL20 (h² = 0.16 and 0.22), align with our broader observation that meal distribution traits are more heritable than feed intake traits during cooler hours.

The similarities between heritability estimates observed in our study and those reported previously confirm that the behavioral traits studied here may serve as indicators of feeding resilience, with notable selection potential, while respecting the physiological and productive constraints of the production system. Interestingly, traits related to meal patterns appear to have higher heritability than those directly related to the amount of feed consumed, which is not surprising as very different feeding patterns can result in similar daily feed intake. For example, Howie et al. [25] showed that, for rather similar daily feed intake (124 g vs. 115 g), two lines of chickens had a very different daily meal number (9.8 vs. 15.3).

### Correlation between indoor feeding behavior traits and outdoor FCR

Beyond exploring the genetic parameters of feeding behavior, our study’s main goal was to better understand the genetic correlations between feeding behavior in IN and FCR in OUT, to optimize indirect selection for FCR in commercial conditions. For that purpose, we focused on identifying heritable behavioral traits (h²> 0.10) in IN that are genetically correlated with FCR in OUT.

The genetic correlations between feeding behavior traits and FCR revealed a complex network of interactions. Some traits positively correlated with FCR were negatively correlated with each other, creating antagonisms that can hinder the efficiency of selection on individual traits. As a result, single-trait selection may not achieve optimal genetic improvement. The genetic variance-covariance matrix (**G** matrix) considers simultaneously all genetic relationships, enabling a more balanced and effective selection strategy [34, 43]. A multi-trait approach that incorporates these correlations can better manage trait interactions, maximizing gains in feed efficiency while minimizing unwanted side effects. Accordingly, we analyzed the **G** matrix of our measured feeding behavior traits to identify key genetic axes, summarizing these complex relationships between multiple traits, which could be integrated into selection programs. Selecting for low **g**_**max**_ (first axis explaining 56% of the genetic variance in feeding behavior), would improve feed efficiency by favoring animals that adjust their feeding patterns, eat more in the morning, less in the evening, and have fewer meals overall. Selecting for high **g**_**2**_ (second axis, 18% of the genetic variance in feeding behavior) would also improve feed efficiency by increasing morning feed intake while maintaining a consistent proportion of morning consumption, as well as reducing the influence of temperature fluctuations on feeding behavior.

Both axes highlight the importance of increasing morning intake and maintaining consistent meal frequency and total consumption. A lower number of meals indicates more efficient regulation of feed intake, improving nutrient utilization and minimizing feeding-related stress [44]. Increased morning consumption, driven by a natural appetite peak after overnight fasting, facilitates efficient digestion and nutrient absorption during active hours, promoting better metabolic regulation [45]. Reducing evening intake further optimizes digestion during the night, preventing disruptions and overfeeding [45]. Feeding time variability reflects behavioral plasticity, which allows animals to adapt their feeding schedules to changing environmental conditions, such as temperature or food availability, further supporting metabolic efficiency. The **g**_**2**_ axis driven by early feeding behavior and by correlations between temperature and meal number, highlights the key role of early morning feeding and the importance of behavioral plasticity in regulating feed intake during warmer periods, thereby limiting unnecessary energy expenditure.

Despite enabling a more precise picture of the complex relationships among traits, multivariate analyses also require powerful designs and a higher number of phenotypes measured. In our study, we used over 500 animals in which we measured 16 traits analyzed using 6 × 6 multivariate models. We must therefore be cautious not to overinterpret our results, and it will be important to replicate these results in a different population.

### Comparison of different selection scenarios

The results obtained in this study showed that the **g**_**max**_ and **g**_**2**_ axes, heritable and correlated with FCR in OUT, can help improve feed efficiency, particularly in our conditions where the heritability of FCR IN was low. Selection on these behavioral traits was estimated to be twice as efficient as current methods of selecting on FCR measured indoors, in terms of expected genetic gain on FCR in outdoor conditions.

Nevertheless, direct selection for FCR in OUT would yield a higher genetic gain than indirect selection of **g**_**max**_ or **g**_**2**_ in IN (53 and 44%, respectively). However, selection for FCR in OUT is currently only possible through collateral selection, to protect the breeding population from sanitary risks on an outdoor range. Selection on collaterals increases the cost of selection and results in a loss of genetic gain usually estimated around 30% (Table 13.3 in [9]). This would considerably reduce the advantage of directly selecting on FCR in OUT. Therefore, selecting on feeding behavior in IN conditions offers a promising approach for improving feed efficiency, while maintaining the sanitary status of breeders.

It should be noted that the genetic parameters estimated here were obtained within a pure line, and the extent to which the expected selection responses would be transmitted to commercial crossbreds remains unknown. Crossbreeding and the generational distance between selection candidates and production birds may attenuate the realized correlated responses, and this warrants investigation in future work.

### Effect on other outdoors production traits

Genetic selection typically requires balancing trade-offs between performance traits such as growth, feed intake, yield, and meat quality. Our results suggest that selection on the **g**_**max**_ axis would positively influence meat quality by reducing acidity (pHu), lightness (L*), and water loss (DL), which are traits often underlying greater meat stability and preservation [46, 47]. This is particularly important in Label Rouge chicken production, in which the meat tends to be more acidic than in conventional production systems [48].

Selection on the **g**_**max**_ axis would have limited effects on carcass composition traits such as breast yield, thigh yield, and abdominal fat. Moreover, moderate correlations between **g**_**max**_ in IN and feed intake in OUT, and between **g**_**max**_ in IN and weight gain in OUT, suggest that selecting for lower **g**_**max**_ could lead to decreased feed consumption and would tend to increase weight gain. However, the overall effect remains uncertain due to the large standard errors associated with the genetic correlations of **g**_**max**_ with both WG and FI. During periods of summer heat stress, chickens often reduce their feed intake due to thermal discomfort, which slows their growth; higher weight gain could therefore help maintain production targets under these conditions. However, the relationship with heat stress is not straightforward, as heavier birds are also more prone to heat stress, which could in turn compromise welfare [49], although this risk may be attenuated in our studied slow-growing breed. Outside warm seasons, increased weight gain could lead to animals exceeding Label Rouge weight requirements [3, 50]. Moreover, during avian influenza episodes, free-range poultry farms have to rapidly switch to confinement rearing for sanitary reasons. During confinement periods, animal mobility is restricted, their weight gain increases and it can lead to exceeding regulatory weight limits [51, 52]. Introducing **g**_**2**_ into the selection scheme would also decrease FI, with unclear effects on WG given the uncertainty around their genetic correlation (r_g_ = -0.19 ± 0.26). However, it may negatively affect meat quality and carcass traits by decreasing meat pHu and breast yield while increasing paleness and abdominal fat. These antagonistic effects on meat quality and carcass traits are of similar magnitude to the unfavorable effects of **g**_**max**_ selection on weight gain, and warrant the same caution in interpretation. In Label Rouge production, where meat quality is a defining market requirement, the trade-offs associated with **g**_**2**_ selection would need to be carefully evaluated before implementation. Future work incorporating index selection across multiple traits would help quantify the net benefit of including **g**_**2**_ alongside **g**_**max**_, and determine whether its contribution to FCR improvement justifies the associated costs in meat and carcass traits.

## Conclusions

Our study identified several behavioral traits, such as meal frequency and morning feeding behaviors, as promising selection targets due to their heritability and genetic correlation with FCR in commercial free-range rearing conditions. In particular, the **g**_**max**_ axis appears to be a prime candidate of selection for improving feed efficiency, meat quality, and maintaining carcass composition. Thus, this study paves the way for breeding strategies that include feeding behaviors to optimize feed efficiency and address challenges related to animal welfare and the sustainability of poultry production. Nevertheless, the relationships between feeding behavior and performance remain complex, as shown by both genetic correlations and genetic analysis of the **G** matrix, and need to be explored in others environments and strains.

## Supplementary Information

Below is the link to the electronic supplementary material.

Supplementary Material 1: Additional files. Additional file 1 Figure S1 Format: word Title: Visits interval to define separate meals threshold by ageDescription: This file presents the time threshold (in seconds) used to distinguish separate feeding meals in chickens, calculated as a function of age. The threshold corresponds to the maximum interval between two feeder visits to consider them part of the same meal. The mean threshold across all ages is 370.68 s

## Data Availability

Data are available at Chaumont, Salomé; Mignon, Sandrine V.; Le Bihan-Duval, Elisabeth; Collin, Anne; Fagnoul, Frederic; Guettier, Elodie; Collet, Julie, 2026, “Data for: Selecting feeding behavior would enhance feed efficiency in slow-growing free-range broiler”, https://doi.org/10.57745/JBTP3U.
