# Supplementary material for: Selecting feeding behavior would enhance feed efficiency in slow-growing free-range broiler
Source: Genet Sel Evol. 2026 Nov 13;58:91. doi: 10.1186/s12711-026-01108-y (PMC13761128; doi:10.1186/s12711-026-01108-y)
Supplement: Supplementary file 1 [file 12711_2026_1108_MOESM1_ESM.docx]

# Visits interval to define separate meals threshold by age

|  | Limite_25S |
| --- | --- |
| 15 | 431 |
| 16 | 369 |
| 17 | 520 |
| 18 | 612 |
| 19 | 654 |
| 20 | 437 |
| 21 | 506 |
| 22 | 506 |
| 23 | 475 |
| 24 | 398 |
| 25 | 354 |
| 26 | 453 |
| 27 | 378 |
| 28 | 405 |
| 29 | 325 |
| 30 | 453 |
| 31 | 362 |
| 32 | 238 |
| 33 | 201 |
| 34 | 237 |
| 35 | 246 |
| 36 | 362 |
| 37 | 491 |
| 38 | 415 |
| 39 | 525 |
| 40 | 588 |
| 41 | 512 |
| 42 | 463 |
| 43 | 392 |
| 44 | 274 |
| 45 | 324 |
| 46 | 350 |
| 47 | 369 |
| 48 | 463 |
| 49 | 461 |
| 50 | 591 |
| 51 | 510 |
| 52 | 391 |
| 53 | 392 |
| 54 | 383 |
| 55 | 419 |
| 56 | 398 |
| 57 | 327 |
| 58 | 317 |
| 59 | 296 |
| 60 | 297 |
| 61 | 336 |
| 62 | 306 |
| 63 | 350 |
| 64 | 322 |
| 65 | 374 |
| 66 | 320 |
| 67 | 342 |
| 68 | 329 |
| 69 | 248 |
| 70 | 335 |
| 71 | 246 |
| 72 | 407 |
| 73 | 344 |
| 74 | 324 |
| 75 | 347 |
| 76 | 268 |
| 77 | 770 |
| 78 | 593 |
| 79 | 620 |
| 80 | 643 |
| 81 | 638 |

**Figure S1** Time threshold (in seconds) used to distinguish separate feeding meals in chickens, calculated as a function of age. The threshold corresponds to the maximum interval between two feeder visits to consider them part of the same meal. The mean threshold across all ages is 370.68 seconds.
